# Integrated Metabolomics and Transcriptomics Analyses Reveal the Metabolic Differences and Molecular Basis of Nutritional Quality in Landraces and Cultivated Rice

**DOI:** 10.3390/metabo12050384

**Published:** 2022-04-22

**Authors:** Zhonghui Zhang, Feng Zhang, Yuan Deng, Lisong Sun, Mengdi Mao, Ridong Chen, Qi Qiang, Junjie Zhou, Tuan Long, Xuecheng Zhao, Xianqing Liu, Shouchuang Wang, Jun Yang, Jie Luo

**Affiliations:** 1 College of Tropical Crops, Hainan University, Haikou 570228, China; zhonghui.zhang@hainanu.edu.cn (Z.Z.); yuandeng@hainanu.edu.cn (Y.D.); lisong.sun@hainanu.edu.cn (L.S.); mengdi.mao@hainanu.edu.cn (M.M.); ridong.chen@hainanu.edu.cn (R.C.); qiangqi@hainanu.edu.cn (Q.Q.); sand_zhou@hainanu.edu.cn (J.Z.); longtuan2001@hainanu.edu.cn (T.L.); xczhao@hainanu.edu.cn (X.Z.); liuxq@hainanu.edu.cn (X.L.); shouchuang.wang@hainanu.edu.cn (S.W.); 2 National Key Laboratory of Crop Genetic Improvement, National Center of Plant Gene Research (Wuhan), Huazhong Agricultural University, Wuhan 430070, China; zhangfeng@mail.hzau.edu.cn; 3 Sanya Nanfan Research Institute of Hainan University, Hainan Yazhou Bay Seed Laboratory, Sanya 572025, China

**Keywords:** rice (*Oryza sativa* L.), metabolome, transcriptome, landrace, anthocyanins, UDP-glucosyltransferase

## Abstract

Rice (*Oryza sativa* L.) is one of the most globally important crops, nutritionally and economically. Therefore, analyzing the genetic basis of its nutritional quality is a paramount prerequisite for cultivating new varieties with increased nutritional health. To systematically compare the nutritional quality differences between landraces and cultivated rice, and to mine key genes that determine the specific nutritional traits of landraces, a seed metabolome database of 985 nutritional metabolites covering amino acids, flavonoids, anthocyanins, and vitamins by a widely targeted metabolomic approach with 114 rice varieties (35 landraces and 79 cultivars) was established. To further reveal the molecular mechanism of the metabolic differences in landrace and cultivated rice seeds, four cultivars and six landrace seeds were selected for transcriptome and metabolome analysis during germination, respectively. The integrated analysis compared the metabolic profiles and transcriptomes of different types of rice, identifying 358 differentially accumulated metabolites (DAMs) and 1982 differentially expressed genes (DEGs), establishing a metabolite–gene correlation network. A PCA revealed anthocyanins, flavonoids, and lipids as the central differential nutritional metabolites between landraces and cultivated rice. The metabolite–gene correlation network was used to screen out 20 candidate genes postulated to be involved in the structural modification of anthocyanins. Five glycosyltransferases were verified to catalyze the glycosylation of anthocyanins by in vitro enzyme activity experiments. At the same time, the different mechanisms of the anthocyanin synthesis pathway and structural diversity in landrace and cultivated rice were systematically analyzed, providing new insights for the improvement and utilization of the nutritional quality of rice landrace varieties.

## 1. Introduction

Rice (*Oryza sativa* L.) is one of the most globally important crops, feeding more than half of the world’s population, thus making it an economically important food source [1]. With the rapid development of agricultural technology and breeding, traditional traits such as rice yield and disease resistance have been greatly improved [2,3,4]. As the traditional traits improve, research has begun to focus on enhancing the nutritional quality of rice [5,6]. Compared with cultivated rice varieties, landraces with specific agronomic traits provide valuable genetic resources for rice genetic improvement and new variety breeding [7]. In particular, landrace varieties, traditionally chosen by farmers of specific regions during the domestication of agriculture, are adapted to specific environments and have the advantages of stress resistance, yield stability, and nutrient richness [8]. Seeds of landraces contain many different colors, such as white, red, and black. The coloring of red and black rice seeds comes from procyanidins and anthocyanins in the pericarp and seed coat of the kernel. Cyanidin-3-*O*-glucose (C3G) and peonidin-3-*O*-glucose (P3G) are usually the predominant components, while other anthocyanin species can be present in little amounts [9,10]. Compared to cultivar rice, landrace such as red and black rice are rich in various nutrients, including anthocyanins, vitamins, flavonoids, and certain essential amino acids. [9,11,12]. Black rice is considered as a panacea for health and longevity in China [10]. Red rice can delay aging and protect against noncommunicable diseases [13], including cancer, cardiovascular disease, diabetes, and metabolic syndrome [14]. Therefore, it is beneficial to analyze the composition of the seed metabolome of landrace varieties and discover the genes that determine their nutritional quality, improving the overall nutritional value of rice.

With the continuous development of metabolomic technology, especially high-throughput metabolite detection technology [15], metabolic detection has been applied to economically significant plants such as rice, corn, wheat, and tomatoes. Corresponding metabolome databases have been established successively due to the previously mentioned technological improvements [16,17,18]. Understanding the nutritional composition and quality improvement of crops provides a theoretical basis and resources [16]. In recent years, there have been reports of a comparative metabolomic analysis using different types of tomato varieties. The comparative metabolomic analysis of 398 tomato populations, including wild tomatoes, heirloom, and modern cultivated tomatoes, found that citric acid can enhance the tomato flavor, malic acid reduces the overall flavor, and geranial alcohols can enhance the sweetness of the fruit [17]. In addition, through tomato breeding, the content of glycoalkaloids in modern cultivated tomatoes was greatly reduced, and the nutrients such as flavonoids and vitamins also decreased compared with wild tomatoes [18]. A rice metabolism database covering the entire growth period of a plant provides a basis for systematically understanding the dynamics and changes of the rice metabolome throughout a growing season [19]. Previously, a metabolomic analysis of various tissues in rice showed significant differences in the accumulation of 90 flavonoids and 16 phenolamides in rice seedlings, leaves, culms, panicles, and roots [20,21]. However, the metabolomic analysis of rice landraces has not been systematically studied, and a comparative metabolomic analysis of landraces and cultivated rice will be helpful to elucidate the key metabolites that determine the nutritional quality of rice.

Breakthroughs in various high-throughput technologies have made it possible to obtain genomic, transcriptomic, and metabolomic data at unprecedented speeds and low costs. Metabolomic and transcriptomic data can be integrated using a correlation-based analysis, a powerful genetic tool for identifying novel genes regulating specific metabolic pathways. Previous studies have indicated that a transcriptional metabolite correlation analysis in potatoes and hundreds of significant pairwise correlations provide clues for tuber nutritional metabolites [22,23]. Multispecies transcriptomic and metabolic analyses were performed on *Mirabilis jalapa* to identify the candidate genes involved in betaine biosynthesis [24]. Eight genes of the colchicine alkaloid biosynthesis pathway are discovered and determined by a combination of transcriptomics, metabolic logic, and pathway remodeling [25]. A falcarindiol biosynthetic gene cluster was discovered in tomatoes, which was directly resistant to fungal and bacterial pathogens, which was directly resistant to fungal and bacterial pathogens with untargeted metabolomics and RNA-sequencing [26]. Meanwhile, some studies indicate that co-expression and a correlation analysis based on transcriptome and metabolome have become one of the most efficient methods for discovering the metabolic pathway and identifying some novel genes [27,28,29,30,31]. With the development of next-generation sequencing technologies, high-density physical or genetic linkage maps based on high-quality genomes have enabled a ‘multi-omics’ analysis that integrates genomes, transcriptomes, and metabolomes [32,33,34,35]. The development of ‘omics’ technology has made it possible to analyze the composition of the nutritional quality and the genetic and biochemical basis of landraces.

In this study, we collected 114 rice germplasm resources from all over China, including 35 landrace and 79 cultivated rice varieties, to systematically compare the nutritional quality differences in seeds between modern rice cultivars and landraces and mine genes that determine the specific nutritional value traits of landrace. A landrace seed metabolism database, covering important nutritional metabolites such as amino acids, flavonoids, lipids, and vitamins, was established by a broad-targeted metabolomic approach. To further reveal the molecular mechanism of the metabolic differences in the landrace and cultivated rice seeds, four cultivars and six landrace seeds were selected for transcriptome and metabolome analysis during germination, respectively. Through the integrated analysis of the transcriptome and metabolome, 358 differentially accumulated metabolites and 1982 differentially expressed genes related to nutritional quality were screened in these varieties. Twenty candidate genes involved in the metabolic synthesis of anthocyanins were screened by the metabolite–gene correlation network, and five glycosyltransferases were verified by in vitro enzyme activity experiments to catalyze the glycosylation of anthocyanins. This study systematically compares the differences in the metabolic profiles of landraces and cultivated rice and provides new insights for the analysis and utilization of the nutritional quality of rice landraces. 

## 2. Results

### 2.1. Comparative Analysis of Metabolic Profiling between Landraces and Cultivated Rice

**Figure 1:**
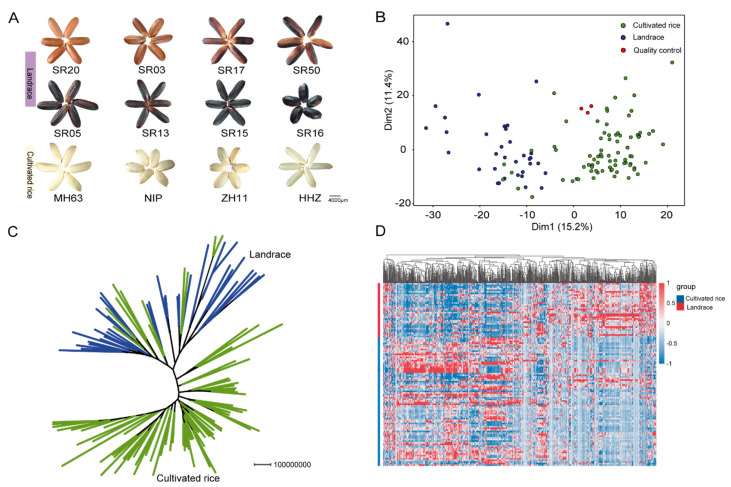
Comparative metabolic differences in cultivar rice and landrace seeds. (**A**) The seed colors between landraces and cultivated rice. The cultivar was represented by white, and the landrace was represented by red and black. (**B**) Principal components analysis (PCA) of the metabolite profiling in different types of rice seeds. Green represents cultivated rice, and red represents quality control, while blue represents landraces. (**C**) Neighbor-joining tree of 114 rice varieties based on 985 metabolites. (**D**) Heatmap based on the metabolome data of cultivated and landrace rice seeds. Red indicates a high abundance, and blue indicates low relative abundance metabolites.

To explore the metabolic basis of the nutritional quality of rice landraces and cultivars, we collected 114 rice core germplasm resources from all over China, including 79 cultivated rice and 35 landraces (Table S1). Compared with cultivated rice, landraces showed significant differences in many important agronomic traits, such as seed color and plant height (Figure 1A and Figure S1). We constructed a rice seed metabolome database using a broad-targeted metabolomic approach with landraces and cultivated varieties, including 985 high-quality metabolic signals. The structures of 549 metabolites were identified by standards and databases, including 85 amino acids and their derivatives, 93 lipids, 25 anthocyanins, 120 flavonoids, and 25 vitamins and their derivatives (Table S2). The principal component analysis (PCA) results showed that landrace and commonly cultivated rice could be divided into two clades, which means that there is a very significant difference in the metabolite compositions between the two types of rice (Figure 1B). Similarly, the metabolome-based phylogenetic tree also divided landraces and cultivars into two clusters, with the results also showing the population structure of 114 rice germplasm resources (Figure 1C). Additionally, the heatmap result showed that there were some accumulation differences in the seeds of landrace and cultivated rice (Figure 1D). The classification results showed that the metabolites accumulated significantly differently between cultivated rice and landrace were mainly flavonoids, vitamins, amino acids, and their derivatives (Figure S2).

Ninety significantly different metabolites in seeds between landraces and cultivated rice were identified. Notably, the contents of anthocyanins and flavonoids were more enriched in landrace seeds than cultivated rice seeds (Figure S3A). The difference folds of peonidin 3, 5-*O*-diglucoside were 4.40, and the difference folds of between naringenin 7-*O*-glucoside and apigenin 6-*C*-glucoside were 9.31 and 9.27, respectively (Table S4). This indicates that there may be some differences in the anthocyanin and flavonoid synthesis pathway between landrace and cultivated rice seed.

### 2.2. Analysis of DAM and DEG in Seeds of between Landrace and Cultivated Rice during Germination

**Figure 2:**
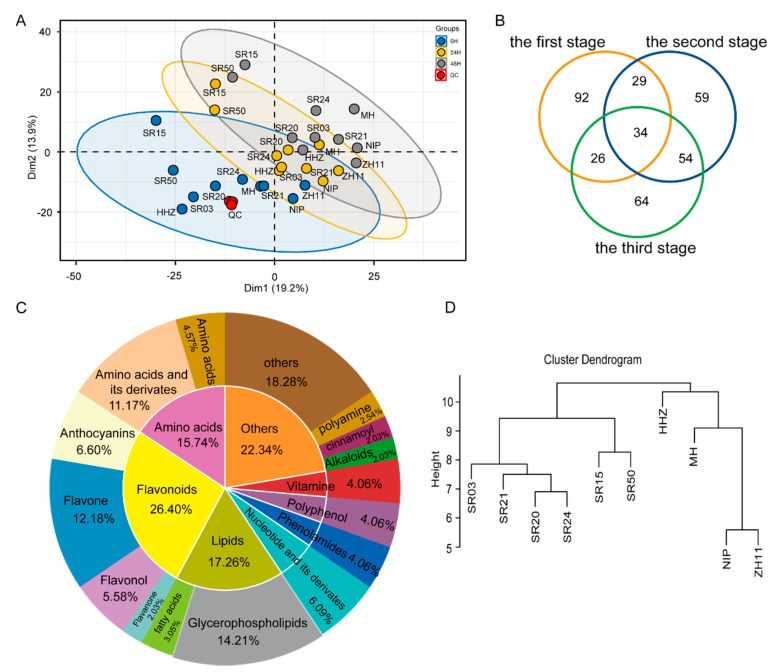
Comparative metabolic analysis in cultivar rice and landrace seeds during germination. (**A**) PCA of the metabolite profiling in seeds of landraces and cultivated rice during germination. (**B**) Venn diagram depicting the number of differential metabolites in the seeds of landraces and cultivated rice during germination. (**C**) The composition and proportion of different metabolites in the seeds of landraces and cultivated rice during germination. (**D**) Phylogenetic tree based on differential accumulated metabolites in seeds of landraces and cultivated rice during germination.

To further reveal that the metabolic difference in the seeds of landraces and cultivated rice, a metabolomic analysis was performed on four cultivars and six landraces at different germination stages. The PCA results showed that seed samples of the three germination stages were divided into three clusters, and each region had the same color distribution (Figure 2A), indicating that the metabolite accumulation is different in seeds with different colors during germination. We calculated the contribution of all metabolites to PC1 and found that the most of lipids and flavonoids contributed more (Table S5). This indicated that there were differences in the accumulation patterns of the lipids and flavonoids during the germination of seeds with different colors. By comprehensively calculating the contribution rate of all metabolites in the principal component analysis, it was found that amino acids, such as valine, threonine, leucine, and isoleucine, had more significant contributions (Figure S3B). This indicates that the accumulation pattern of amino acids changed significantly during seed germination.

A differential fold analysis of the metabolite content in these seeds during germination indicates that a total of 358 differential metabolites (DAMs) were found during seed germination, of which 34 metabolites were differentially accumulated at three stages of seed germination, 92 metabolites were differentially accumulated at the first stage, 59 metabolites were differentially accumulated in the second stage, and 64 metabolites were differentially accumulated at the third stage (Figure 2B). These DAMs mainly included amino acids, lipids, vitamins, and flavonoids (Figure 2C). In detail, amino acids and flavonoids were mainly accumulated at the first stage and lipids and anthocyanins were mainly accumulated at the second and third stages (Table S6). These results indicated that there were differences in the metabolite accumulation patterns at different stages during seed germination. To further verify whether the accumulation of differential metabolites conforms to the distribution law during seed germination, a phylogenetic analysis based on the differential accumulation metabolites of seeds at the first stage showed that landraces and cultivated rice were clustered into two clades, and this result was consistent with that in Figure 2D. These results indicate that the seed color may be the cause of metabolic differences between cultivars and landraces during seed germination.

**Figure 3:**
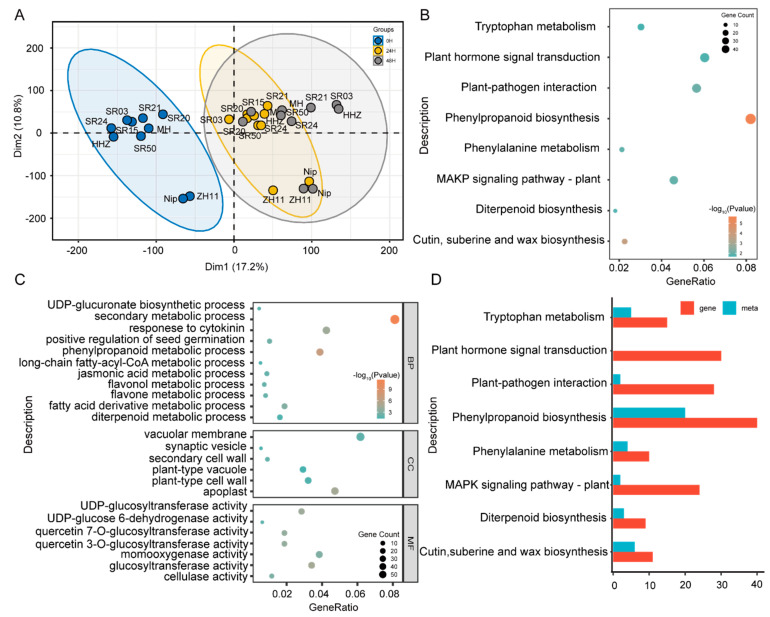
Construction of a metabolite–gene regulatory network in cultivar rice and landrace seeds during germination. (**A**) A PCA analysis based on the transcriptome in the seeds of landraces and cultivated rice during germination. (**B**) KEGG analysis of DEGs in the seeds of landraces and cultivated rice during germination. *Y*-axis represents KEGG pathways, and *X*-axis indicates the gene ratio. (**C**) GO enrichment analysis of DEGs in the seeds of landraces and cultivated rice during germination. MF, molecular function; BP, biological process; CC, cellular component. (**D**) KEGG analysis of DAMs and DEGs in the seeds of landraces and cultivated rice during germination.

Accordingly, we sequenced the transcriptomes of four cultivars and six landraces from three different germination stages, respectively. A PCA analysis showed that landraces were mainly in the direction of principal component 1; meanwhile, there were obvious indica–japonica differences in the cultivars in the direction of principal component 2, indicating that the landraces clustered with MH and HHZ may belong to indica (Figure 3A). Furthermore, the results of the Gene Ontology (GO) enrichment analysis displayed that genes with greater PCA contributions were mainly related to the processes of amino acids, lipids, vitamins, and flavonoids (Figure S4). These results suggest that these genes may be involved in differential metabolite metabolism in the seeds of landraces and cultivated rice during germination.

To further verify the above results, we performed a differential expression analysis and screened a total of 1982 differentially expressed genes (DEGs) (Table S7). Meanwhile, the results of the KEGG enrichment analysis and GO analysis were performed on DEGs. The KEGG analysis results showed that the DEGs were mainly involved in amino acid and flavonoid synthesis pathways (Figure 3B). Moreover, the GO analysis results also displayed that the DEGs were mainly related to the metabolisms of amino acids and flavonoids (Figure 3C). Further analysis found that the main DEGs were mainly enriched in the phenylpropane metabolism and amino acid metabolism (Figure 3D). These results indicate that DAMs and DEGs maybe synergistically influence seed germination in landraces and cultivated rice.

### 2.3. Correlation Network Based on Genes and Metabolites during Seed Germination

**Figure 4:**
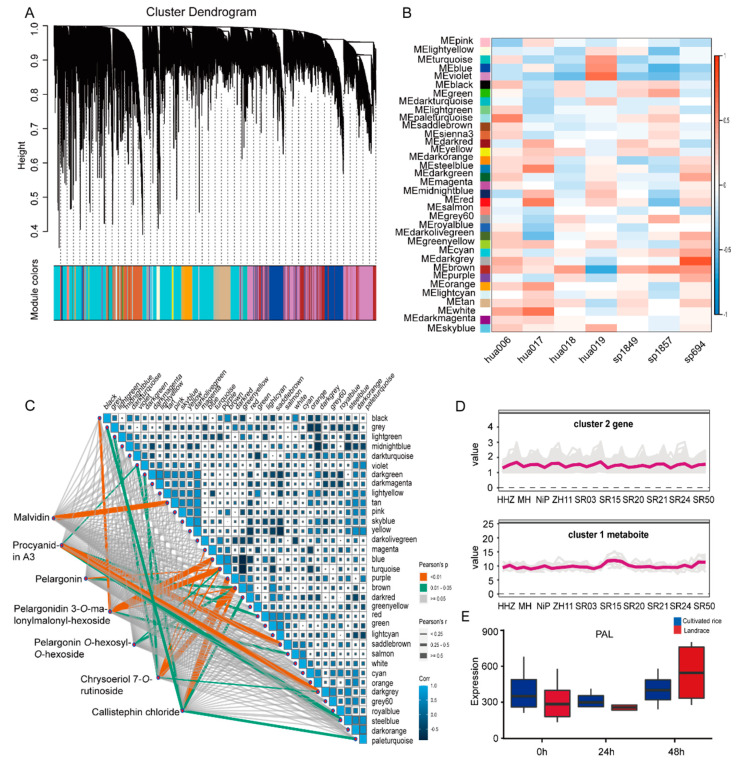
Correlation network analysis based on metabolites and genes in the seeds of landraces and cultivated rice during germination. (**A**) Construction of a co-expression gene module. The same color represents the same module. If the module feature genes between two different modules are similar, they will be merged automatically. (**B**) Heatmap between metabolites and 35 gene modules. *Y*-axis represents each module, and the *X*-axis represents each trait. Red shows upregulated transcripts, and blue shows downregulated transcripts. (**C**) Co-expression network of genes in modules. (**D**) Dynamic of metabolite accumulation and gene expression in co-expression clusters. Metabolite and gene clusters are represented by red. The numbers of the *X*-axis indicate the germination stage. (**E**) The expression level of *PAL* in the seeds of landraces and cultivated rice during germination.

To decipher the molecular basis of differential metabolites in the seeds of landraces and cultivated rice during germination, the WGCNA based on transcriptome data during rice seed germination showed that the transcriptome data were divided into 35 different gene modules (Figure 4A). The genes in each module were significantly related and could jointly regulate the same metabolic pathway. Further, a correlation network analysis based on the differential genes and metabolites during rice seed germination displayed that some metabolites of the phenylpropanoid pathway, especially some flavonoids and anthocyanins, were positively correlated with each gene module (Figure 4B). Moreover, malvidin and procyanidin A3 were highly correlated with these genes (Figure 4C and Table S9). These results suggest that these genes may be involved in anthocyanin biosynthesis in the seeds of landraces and cultivated rice.

Meanwhile, we performed a cluster analysis based on the gene expression and anthocyanin accumulation at different germination stages to further screen candidate genes and obtained 12 gene clusters and 6 metabolite clusters (Figures S5 and S6). The comparative analysis displayed that the gene expression pattern in cluster 2 was consistent with the accumulation pattern of peonidin, delphinidin, and cyanidin in cluster 1 (Figure 4D). Many reported genes of anthocyanin synthesis in cluster 2, such as CHI (Chalcone isomerase), PAL (Phenylalanine ammonia lyase), and 3GT (Anthocyanin 3-*O*-glucosyltransferase), were found. Their transcription levels, especially *PAL*, were distinctly higher in the seeds of landraces at the third stage (Figure 4E), which means that the genes in cluster 2 may regulate flavonoid and anthocyanin accumulation in the seeds of landraces.

### 2.4. The Metabolic Diversity of Anthocyanins and Its Molecular Mechanism in Different Types of Rice Seeds

**Figure 5:**
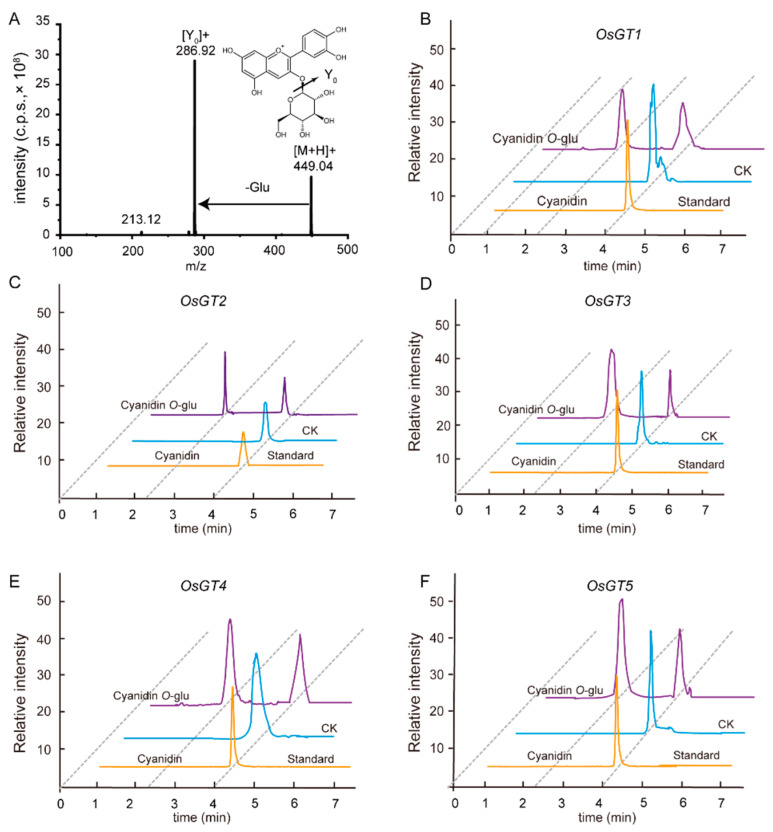
Analysis of the in vitro enzyme activity of OsGTs. (**A**) MS/MS spectra information of cyanidin-3-*O*-glucoside with the authentic standard. The reactions were performed with 5 purified OsGTs, UDP-glucose, and cyanidin as substrates: GT1, glucosyltransferase 1 (**B**); GT2, glucosyltransferase 2 (**C**); GT3, glucosyltransferase 3 (**D**); GT4, glucosyltransferase 4 (**E**); GT5, glucosyltransferase 5 (**F**).

Twenty candidate genes encoding glycosyltransferases (GTs) were screened from gene cluster 2 by the gene–metabolite correlation network, combined with the mGWAS results reported [16]. These glycosyltransferases may have contributed to the difference of anthocyanin accumulation in the seeds of landraces and cultivated rice (Table S9). To verify the function of these genes, we selected five GTs and constructed corresponding protein expression vectors. The results of in vitro enzyme activities showed that LOC_Os07g32630 (GT1), LOC_Os02g37690 (GT2), LOC_Os06g18140 (GT3), LOC_Os06g18010 (GT4), and LOC_Os07g32620 (GT5) could all catalyze cyanidin as a substrate to form cyanidin *O*-glucoside, whereas their catalytic activities for cyanidin were different (Figure 5A–F). Additionally, GT2 used malvidin and procyanidin B2 as substrates to produce malvidin *O*-glucoside and procyanidin B2 *O*-glucoside, respectively (Figure S7A,B). GT5 used peonidin and procyanidin A1 as substrates to form the glycosylation of peonidin and procyanidin A1 (Figure S7C,D), while GT4 catalyzed malvidin, peonidin, and procyanidin A1 for glycosylation (Figure S7D,E). In all, the results suggest that all five GTs have the functions of anthocyanin glycosyltransferase.

**Figure 6:**
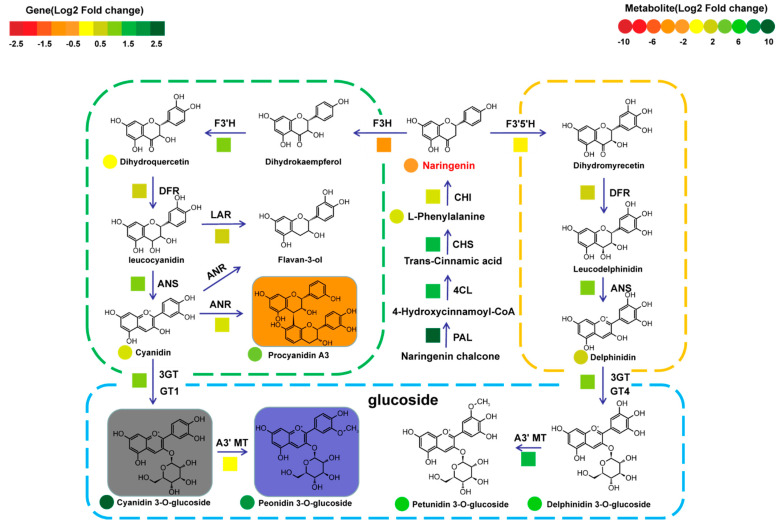
Integrated transcriptomic and metabolomic data to exhibit the anthocyanin synthesis pathway in the germination seeds of cultivar rice and landrace. A heatmap represents the folds of the expression levels of the corresponding structural genes in the seeds of landraces and cultivated rice, and from red to green in the heatmap indicates the expression levels of structural genes ranging from low to high. Genes in anthocyanin synthesis are shown as follows: *PAL*, phenylalanine ammonia lyase; *C4H*, cinnamate 4-hydroxylase; *4CL*, 4-coumarate CoA ligase; *CHS*, chalcone synthase; *CHI*, chalcone isomerase; *F3H*, flavanone 3-hydroxylase; *F3′H*, flavonoid 3′-hydroxylase; *F3′5′H*, flavonoid 3′,5′-hydroxylase; *DFR*, dihydroflavonol reductase; *ANS*, anthocyanidin synthase; flavonoid 3-*O*-glucosyltransferase; *3GT*, flavonoid 3-*O*-glucosyltransfers; *GT1*, glucosyltransferase 1; *GT4*, glucosyltransferase 4.

Meanwhile, the gene expression levels and metabolite contents related to anthocyanin synthesis were analyzed in the germination seeds of landraces and cultivated rice. Compared with cultivated rice, the expression levels of *OsCHS*, *OsF3′H*, *OsF3′5′H*, and *Os3GT* were higher than those in most seeds of cultivated rice (Figure S8A–I and Table S11). Accordingly, the contents of cyanidin 3-*O*-glucoside, petunidin 3-*O*-glucoside, and delphinidin 3-*O*-glucoside were also higher than those in most seeds of cultivated rice (Figure S9A–C and Table S12). Additionally, the expression levels of most structural genes and the accumulation of metabolites in anthocyanin synthesis were also upregulated in the seeds of landraces (Figure 6). A comparative analysis based on transcriptome and metabolome showed that anthocyanins and the key genes of anthocyanin synthesis in landraces were generally higher than those in cultivated rice (Figure 6), which revealed the reason for anthocyanin-specific accumulation in landraces.

## 3. Discussion

Rice is one of the most anthropogenically and economically essential crops globally, and improvements of grain yield and disease resistance are the primary goals of crop genetic improvement [36]. In recent years, the improvement and enhancement of rice nutritional quality has received wide attention [37]. Wild rice and landraces with rich genetic diversity have become important starting materials for rice genetic improvement and new variety cultivations [38]. Landraces have been attracting attention due to their local environmental adaptability, abiotic stress tolerance, and specific metabolic components [39]. During continuous domestication and improvement, landraces have displayed some different characteristics with cultivated rice [40].

Our study indicates that there are some differences in metabolite accumulation between landraces and cultivar seeds, especially amino acids, fatty acids, vitamins, and flavonoids (Figure S2). This metabolic difference may be the reason for the differences in the nutritional quality between landraces and cultivars. Some research suggests that the contents of some nutrients in the seeds of landrace are obviously high, and it is good for healthcare [41,42]. A nutritional quality analysis of wheat seeds displays that wheat seeds of different colors have differences in their nutritional quality, especially differences in the accumulation of amino acids and anthocyanins [43,44]. Black rice is rich in anthocyanins, and long-term consumption can play a role in preventing diseases [45,46]. In this study, most landraces collected with colors were fully rich in anthocyanins and amino acids (Figure 1A). Our results were consistent with the published results about black and purple wheat. This indicates that the results obtained in this study are credible.

During the in-depth study of plant metabolomics, it was found that metabolites can be used as a special genetic phenotype to reflect the genetic differences in population materials [47]. Studies indicate that natural populations of rice and tomatoes have obviously genetic differences between different types of materials. Meanwhile, this genetic difference is also reflected in the results of the metabolic difference [48,49,50]. A metabolomic analysis of rice and tomato populations showed that there were differences in metabolite accumulation among materials of different populations [51,52]. Therefore, the differences on a genetic basis can be reflected by metabolic differences. A principal component analysis and phylogenetic analysis based on metabolic data in the cultivar and landrace seeds showed that the cultivar and landraces were clearly divided into two clades (Figure 1B,C). This result not only suggests that there are metabolic differences between cultivars and landraces but also suggests that genetic differences may be responsible for the metabolic differences between landraces and cultivars.

A study indicated that there are obvious phenotypic differences between colored rice and cultivars in the process of plant growth and development [53]. The difference is mainly in the color of rice leaves. For instance, most cultivated rice shows green leaves, while some colored rice shows purple leaves [54]. However, the molecular regulation mechanism of the seed germination in colored rice still remains unknown. Therefore, we selected 10 representative landrace and cultivated rice varieties for seed germination experiments, and we integrated the metabolome and transcriptome analyses to explore the molecular regulation mechanism of seed germination in colored rice. The results showed that there were significant differences in the metabolism and transcriptome between cultivated rice and colored rice during seed germination. Three hundred and fifty-eight differential metabolites and 1982 differentially expressed genes were obtained by differential fold change analyses, respectively. The differential metabolites during seed germination mainly included amino acids, lipids, vitamins, and flavonoids, which were the same as the main differential metabolites among seeds. It indicates that the difference of the nutrient contents among the seeds may indirectly affect the variation trends of nutrient contents among germination seeds.

The GO enrichment analysis and KEGG analysis of the differentially expressed genes showed that the differentially expressed genes were mainly related to the biosynthesis of amino acids and flavonoids (Figure 3B,C). Since there were significant differences in anthocyanin accumulation between the landrace and cultivated seeds, it infers that there were differences in the anthocyanin accumulation of cultivar and landrace seeds during germination. Next, the turquoise module with a high correlation with anthocyanins was obtained by the WGCNA and the reported mGWAS mapping results. Twenty candidate genes were finally screened in the turquoise module, and five candidate genes were verified for cyanidin glycosylation modification in vitro: namely, *OsGT1*, *OsGT2*, *OsGT3*, *OsGT4*, and *OsGT5*, respectively. This indicates that the five candidate genes have a certain universality and provide a new perspective for understanding the structural diversity of anthocyanins.

In order to visualize the differences in anthocyanin synthesis in landrace and cultivar seeds during the germination, the metabolome, and transcriptome analyses in germinated seeds of cultivars and landrace at the first stage displays the anthocyanin accumulation mode in cultivars and landrace seeds during the germination stage (Figure 6). The comparison analysis of the gene expression levels found that the expression level of *F3H*, a key gene of anthocyanin synthesis, in landraces was lower than that in cultivated rice, but the accumulation of anthocyanins in landraces was higher than that in cultivated rice. This may be due to the higher expression of genes involved in anthocyanin glycosylation in landraces than in cultivated rice.

Efficient, precise, and directional molecular design breeding is a new method for cultivating novel rice varieties that can aggregate excellent traits, such as yield, disease resistance, and nutritional quality. This study revealed the nutrient composition of landraces and analyzed the biochemical and genetic basis of landraces, providing abundant genetic resources for molecular design breeding. Metabolome-assisted modern molecular design breeding could potentially provide a new method and theoretical guidance for improving the nutritional quality of rice and cultivating new varieties.

## 4. Materials and Methods

### 4.1. Plant Materials and Growth Conditions

The rice varieties used in this study were from the Metabolic Biology Laboratory of Hainan University, including 79 modern cultivated rice and 35 landrace rice varieties (Table S1). The rice plants were grown in Haikou, Hainan Province during August–November at 2018. Cultivation condition and field management of rice refer to the previous literature reports [49]. Harvested paddy rice was dried and stored at room temperature for seed metabolite testing and seed germination experiments [49].

To study the metabolic differences of the cultivar rice and landrace seeds, we selected 4 cultivars and 6 landraces for seed germination. Two hundred fully filled grains were selected from each variety and were transported to the petri plates (15 cm in diameter) germination [52]. The ten varieties were soaked in water for 24 h in a chamber set at 32 °C and 70% relative humidity in the dark. After, germination of the seeds was checked every 2 h [52]. When the number of broken glume seeds reached 60% of the total number of germinated seeds, the germinated seeds were transferred to new petri plates cultivation (28 °C, 70% relative humidity, darkness). Then, the broken glume seeds were divided into three parts and were cultured for 0 h as the first stage, 24 h as the second stage, and 48 h as the third stage, respectively. We selected 15 seeds of every rice variety as the RNA sample of one biological replicate and another 15 seeds also as a metabolite sample of one biological replicate. Three biological replicates were performed at each time point.

### 4.2. Metabolic Sample Preparation and Profiling

The samples were lyophilized and ground into powder using a mix mill (MM400, Retsch) with a zirconia bead for 1 min at 30 Hz. Powder (100 mg) was weighed and extracted overnight at 4 °C for 12 h with 70% aqueous methanol (methanol: H_2_O, 70:30, *v*/*v*), followed by centrifugation (4 °C, 10,000× *g*, 10 min). The supernatants were collected and filtration (SCAA-104, 0.22 mm pore size; ANPEL, Shanghai, China, http://www.anpel.com.cn/ (accessed on 21 January 2021)) before the LC-MS analysis. The samples were equally mixed into multiple quality control samples for testing the instrument stability [19].

In this study, a seed metabolism database was constructed based on LC-MS, and a combination of targeted metabolic detection and non-targeted metabolic detection was used to detect a metabolic sample. The liquid chromatography conditions were set as follows: the mobile phase consisted of water (0.04% acetic acid, A phase) and acetonitrile (0.04% acetic acid, B phase). The analysis was carried out with an elution gradient as follows: 0 min, 5% B; 0–10 min, 5–95% B; 10–11 min, 95% B; 11–11.1 min, 95–5% B; 11.1–15 min, 5% B. A C18 column (2.1 × 100 mm, 1.9 μm, shim-pack VP-OSD) was used to separate the samples. The column temperature was 40 °C. The autosampler temperature was 4 °C, and the injection volume was 2 μL.

A nontarget metabolic profiling analysis using LC-ESI-Orbitrap-MS/MS was performed in Full MS/dd-MS^2^. Data were acquired using the following settings: the sheath gas flow rate was 40 Arb; the auxiliary gas flow rate was 12 Arb; the capillary temperature was 360 °C; the S-lens voltage was 55 V; the auxiliary gas heating temperature was 350 °C; the ion scanning range was 100–1500 m/z; the full MS resolution was 70,000; the MS/MS resolution was 50,000; the collision energy was 20/40/60 in NCE mode, and the spray voltage was 3000 V. The mix sample was performed in the Full MS/dd-MS^2^ mode by Q Exactive plus Orbitrap LC-MS/MS using Compound Discoverer 3.1 software to analyze the raw data.

Targeted metabolic profiling analysis was done using multiple reaction monitoring using LC-ESI-QTRAP-MS/MS. The ESI source conditions were set as follows: the source temperature was 500 °C; the ion spray voltage was 5500 V; the ion source GSI, GSII, and CUR were set at 55, 60, and 25 psi, respectively, and the CAD was set to high mode. The mix sample was performed in the multiple reaction monitoring (MRM) mode using Analyst 1.7.3 software to analyze the raw data. Each sample was performed in the scheduled multiple reaction monitoring (s-MRM) mode by 6500 QTRAP using Multi Quant 3.0.3 software for the quantification of metabolites. The detection scan window was set to 60 s, and the target scan time was 1.5 s. A total of 986 transitions were monitored, and the original data were processed by Multi Quant 3.0.3. software (Table S3). In order to improve the normalization, the relative signal strength of the metabolite was divided and normalized according to the internal standards (0.1 mg L−1 lidocaine), and log 2 was then used to transform the value [18,32].

### 4.3. Transcriptome Data Analysis

Rice genome and gene information for reference cultivar, Nipponbare (*Oryza sativa* L. subsp. japonica) was downloaded from (ftp://ftp.plantbiology.msu.edu/pub/data/EukaryoticProjects/osativa/annotationdbs/pseudomolecules/version_7.0/all.dir/ (accessed on 10 August 2020)). An Illumina Hi Step 4000 instrument generated raw reads, and the sequencing read length was double-ended 2*150 base pairs (bp) (PE150) and processed with fastq to filter out adapters and low-quality sequences. The clean reads were mapped using HISAT2 in the reference genome. The gene expression level was quantified using the feature counts based on the expected number of transcripts per kilobase of the exon model per million mapped reads (TPM) method. A differential expression analysis was performed between two groups of colored samples using the DESeq2 R package in Bioconductor version 1.30.0 with adjusted *p*-values. The differential expression levels were determined using the Benjamini–Hochberg FDR multiple testing correction with an adjusted *p*-value < 0.05 and |log2fold change| > 1.2, according to the reference [55].

### 4.4. GO and KEGG Enrichment for Differentially Expressed Genes

Functional-enrichment analyses, including Gene Ontology (GO) and the Kyoto Encyclopedia of Genes and Genomes (KEGG), were performed to identify differentially expressed genes (DEGs) that were significantly enriched in GO terms and metabolic pathways at Bonferroni-corrected *p*-values ≤ 0.05 compared with the whole-transcriptome background. GO functional enrichment and a KEGG pathway analysis were carried out using the cluster Profiler R package in Bioconductor version 3.18.0. The enriched GO terms of the biological process, molecular function, and cellular component categories were visualized with a dot plot.

### 4.5. Phylogenetic Analysis and Principal Component Analysis Based on Metabolites

The principal component analysis and the phylogenetic tree were constructed based on 985 metabolites of 114 rice varieties. The pairwise population distance was used to construct a neighbor-joining tree using the software PHYLIP (version 3.69). The software ITOL (https://itol.embl.de/ (accessed on 18 November 2020)) and MEGAX were used for visualizing the phylogenetic tree. A principal component analysis of the metabolites was performed using the Factoextra R package.

### 4.6. Co-Expression Network Analysis for the Construction of Modules

The highly co-expressed gene modules were inferred from the DEGs using a weighted gene co-expression network analysis (WGCNA), an R package, |*r*| ≥ 0.5, and *p*-value < 0.001 as the thresholds for the screening conditions [56,57]. WGCNA network construction and module detection were conducted using an unsigned topological overlap matrix (TOM), a power β of 10, a minimum module size of 30, and a branch merge cut height of 0.25. The module eigengene (the first principal component of a given module) value was calculated and used to evaluate the association of modules with anthocyanin contents in the 30 samples. The X genes in the significant module turquoise were further clustered and refined using the Cluster function in the R package, and the expression trends of genes in different clusters and the accumulation patterns of anthocyanins detected by LC-MS were further analyzed.

### 4.7. Analysis of Gene Expression Levels by qRT-PCR

The samples at one stage during germination were ground into fine powder in liquid nitrogen. The RNA (Ribonucleic Acid) was extracted by Fast Pure Plant Total RNA Isolation Kit (Polysaccharides & Polyphenolics-rich) (VAZYME, Nanjing, China) according to the manufacturer’s protocol. The total RNA (2 µg) was reverse-transcribed to cDNA with TransScript^®^ One-Step RT-PCR SuperMix (TransGen, Beijing, China) according to the manufacturer’s instructions at 42 °C for 30 min and 85 °C for 10 s (s). The reaction system contained 5 μL TB GREEN, 1 μL cDNA template, 0.4 μL forward and reverse primer, and 0.2 μL ROX DYE2. The structural genes in anthocyanin biosynthesis were detected by the quantitative real-time (qRT-PCR). qRT-PCR was conducted on a (AriaMx Real-Time PCR) system under the following program: 95 °C for 3 min and 40 cycles of 95 °C for 20 s, 60 °C for 30 s, and the melting curve was performed after the amplification according to the program: 95 °C 20 s, 65 °C 30 s and 95 °C 30 s. Rice ubiquitin gene (LOC_Os09g39500) was used as an internal reference control. The expression levels genes detected were normalized to ubiquitin and calculated by the 2^−ΔΔCT^ method [58]. Three independently biological replicates were performed for each gene. The primers of the genes were designed by Primer 3 plus, and the sequence information for the primers were provided in the Table S13.

### 4.8. Recombinant Protein Expression and In Vitro Enzyme Assay

We cloned the full cDNAs of OsGT1, OsGT2, OsGT3, OsGT4and OsGT5 from Nipponbare into the pGEX-6p-1 expression vectors (Novagen) with glutathione S-transferase tags. Recombinant proteins were expressed in BL21 (DE3) cells (Novagen) and then were induced by 0.1 mM isopropyl-β-D-thiogalactoside (IPTG) and continued to be incubated for 16 h at 20 °C. Cells were collected and pellets were resuspended in lysis buffer (50 mM Tris-HCl, pH 8.0, 400 mM NaCl). The cells were disrupted by the high-pressure cracker and cell debris was removed by centrifugation (14,000× *g*, 1 h). Glutathione Sepharose 4B agarose (GE Healthcare) was added to the supernatant containing the target proteins. After incubation for 1 h, the mixture was transferred into a disposable column and washed extensively with lysis buffer (five column volumes). Target proteins in collections were confirmed by SDS-PAGE and purified recombinant proteins were used for in vitro enzyme assays. The enzyme reactions in vitro were carried out in a total volume of 100 microliters (μL) with 200 micromoles per liter (μmol L^–1^) flavonoids or anthocyanins as substrates, 1.5 mmol L^–1^ UDP-glucose as donor, 5 mmol L^–1^ MgCl2, and 500 nanograms (ng) of purified protein in Tris-HCl buffer (100 mmol L^–1^, pH 7.4) and were incubated at 37 °C for 20 min. Then the reactions were stopped by 300 μL of ice-cold methanol.

## Data Availability

RNA sequence data that support the findings of this study have been deposited under SRA BioProject accession number PRJNA830550.

## Supplementary Materials

The following are available online at https://www.mdpi.com/article/10.3390/metabo12050384/s1, Figure S1. Glume colors of landrace. Figure S2. Heatmap based on the metabolites of amino acids, flavonoids, lipids, and vitamins in cultivated and landrace seeds. Figure S3. The analysis of metabolites in the germination seeds of cultivated rice and landraces. Figure S4. The GO analysis for genes in germination seeds of cultivated rice and landraces. Figure S5. Dynamic analysis of gene expression in cultivated rice and landraces during seed germination. Figure S6. Dynamic analysis of metabolite accumulation during seed germination. Figure S7. Analysis of in vitro enzyme activity of OsGT1 to OsGT5. Figure S8. Relative expression levels of structural genes in anthocyanin synthesis in germination seeds of cultivated rice and landrace. Figure S9. Relative content of anthocyanins in germination seeds of cultivated rice and landrace. Table S1. The information of rice varieties used in this study. Table S2. Scheduled MRM transition for widely targeted metabolite analysis in landrace seed. Table S3. Data matrix of 985 metabolite signals in 114 rice accessions. Table S4. Differential metabolites in cultivated and landrace seeds. Table S5. Loadings (correlation coefficients between the original variables and the principal components) of three PCs (PC1, PC2, and PC3) for germinated rice sample. Table S6. Differential accumulation of metabolites in germination seeds of landrace and cultivated rice. Table S7. Differentially expressed levels of genes in germination seeds of landrace and cultivated rice. Table S8. WGCNA analysis genes within the gene module. Table S9. Candidate genes screened may be involved in anthocyanin synthesis pathway by WGCNA and mGWAS. Table S10. Candidate genes successfully verified in vitro. Table S11. The relative expression levels of genes related to anthocyanin biosynthesis in germination seeds of cultivated rice and landrace. Table S12. the relative content of anthocyanin in in germination seeds of cultivated rice and landrace. Table S13. The information of primers used for qRT-PCR in this study.
