# Supplementary material for: Integrated Metabolomics and Transcriptomics Analyses Reveal the Metabolic Differences and Molecular Basis of Nutritional Quality in Landraces and Cultivated Rice
Source: Metabolites. 2022 Apr 22;12(5):384. doi: 10.3390/metabo12050384 (PMC9142891; doi:10.3390/metabo12050384)
Supplement: Supplementary file 1 [file metabolites-12-00384-s001.zip › Supplementary Figures.pdf]

Integrated metabolomics and transcriptomics analyses reveal the metabolic differences and molecular basis of nutritional quality in landraces and cultivated rice

Zhonghui Zhang[1, †], Feng Zhang[2,†], Yuan Deng[1,3,], Lisong Sun[1,3,], Mengdi Mao[1], Ridong Chen[1,3,], Qi Qiang[1], Junjie Zhou[1], Tuan Long[1,3,], Xuecheng Zhao[1,3,], Shouchuang Wang[1,3,] , Jun Yang[1,3,*] and Jie Luo[1,3,*]

1 School of Tropical Crops, Hainan University, Haikou, Hainan 570288, China;
2 National Key Laboratory of Crop Genetic Improvement and National Center of Plant Gene Research (Wuhan), Huazhong Agricultural University, Wuhan 430070, China;
3 Sanya Nanfan Research Institute of Hainan University, Hainan Yazhou Bay Seed Laboratory, Sanya, 572025, China.
*Correspondence: yang9yj@hainanu.edu.cn (J.Yang.), jie.luo@hainanu.edu.cn (J. Luo.)

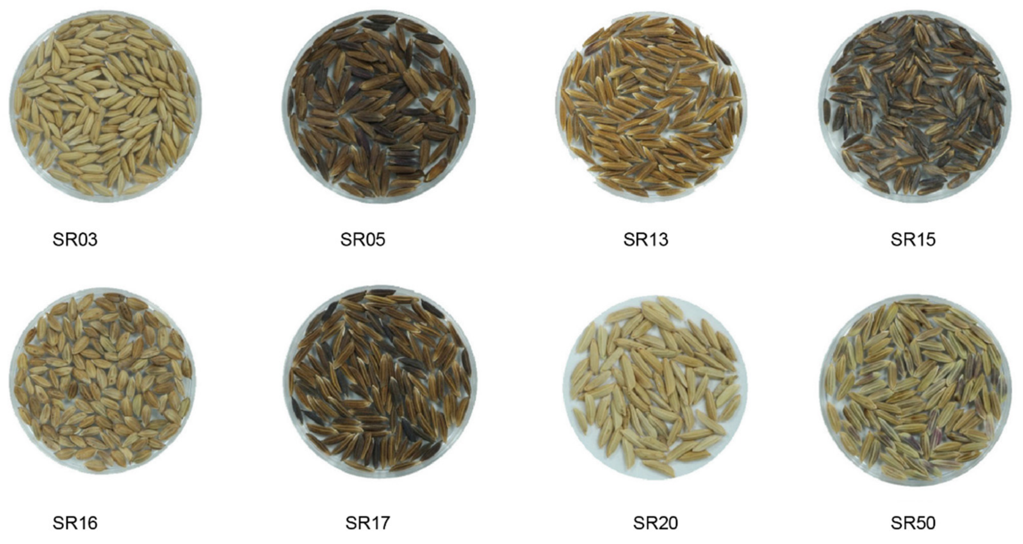

Figure S1. Glume colors of landrace.

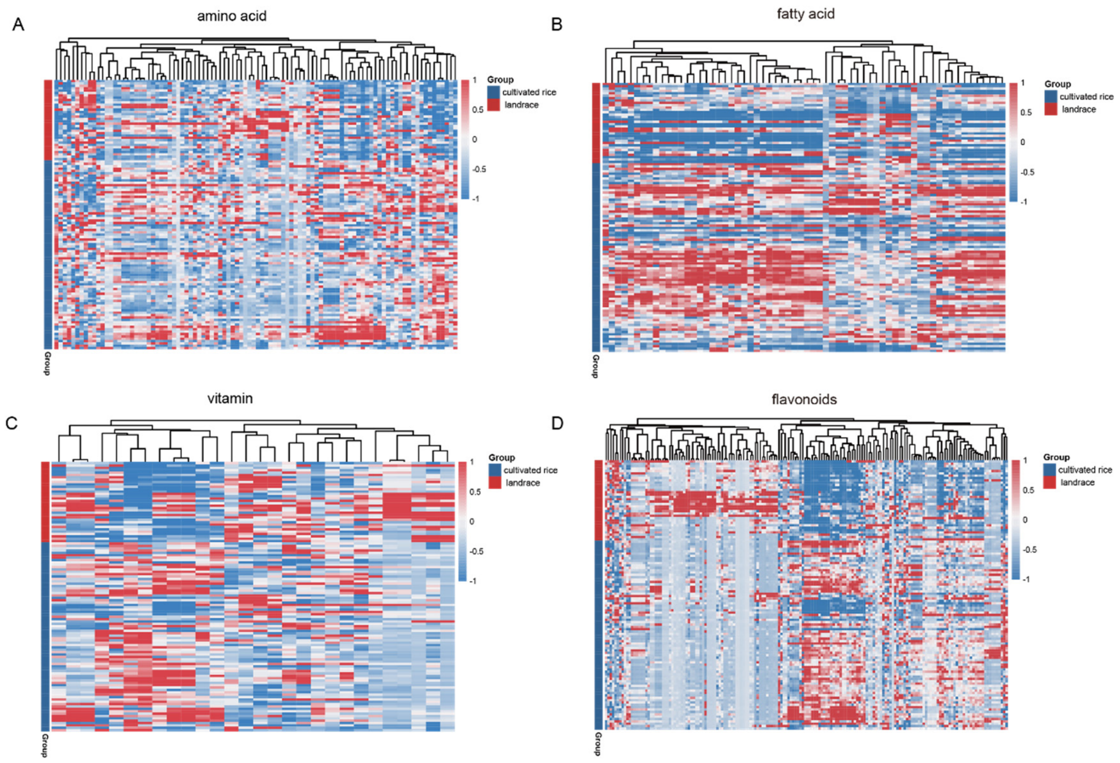

Figure S2. Heatmap based on the metabolites of amino acids, flavonoids, lipids, and vitamins in cultivated and landrace seeds. (A) amino acids; (B) flavonoids; (D) vitamins; for (A-D), red indicates landrace, white cultivated represented by blue. Red indicates high abundance, whereas low relative metabolites are blue.

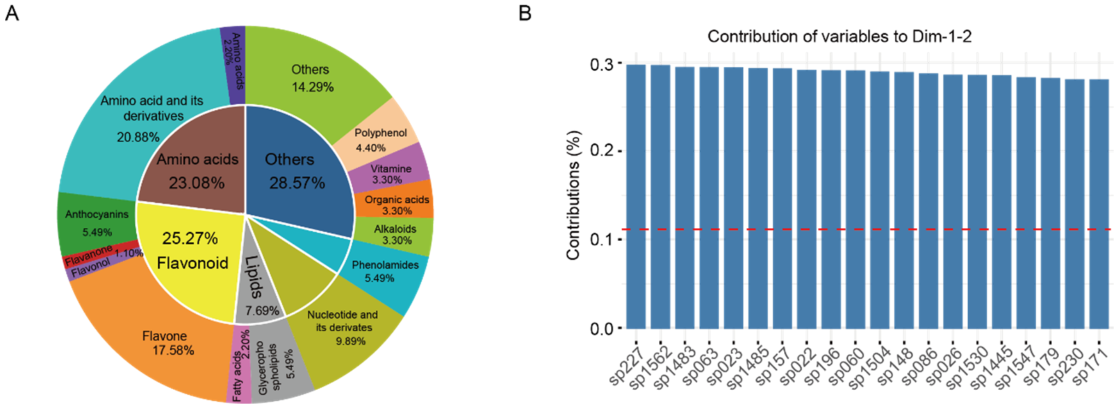

Figure S3. The analysis of metabolites in the germination seeds of cultivated rice and landraces. (A) The composition and proportion of different accumulate metabolites in the cultivated species and landraces. (B) The analysis of the contribution of metabolites to the principal components of seeds at different stages of dehiscence, the top 20 metabolites contributing to principal component 1 and principal component 2. The X-axis refers to different types of metabolites, Y-axis represent the contributions of metabolites.

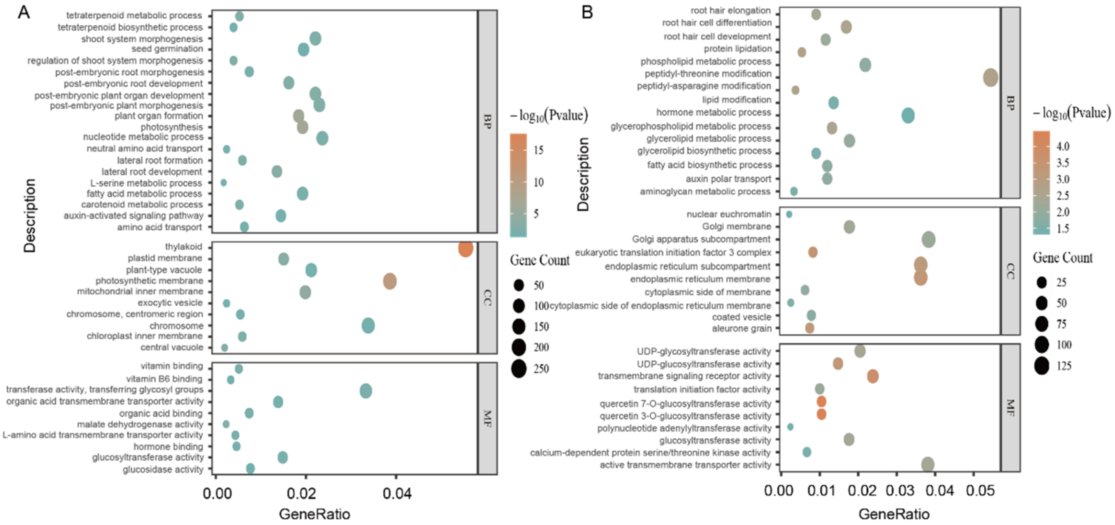

Figure S4. The GO analysis for genes in germination seeds of cultivated rice and landraces. (A) The results of GO analysis for genes with greater than average contribution in Dim1. MF, molecular function; BP, biological process; CC, cellular component. (B) The results of GO analysis for genes with greater than average contribution in Dim2. MF, molecular function; BP, biological process; CC, cellular component.

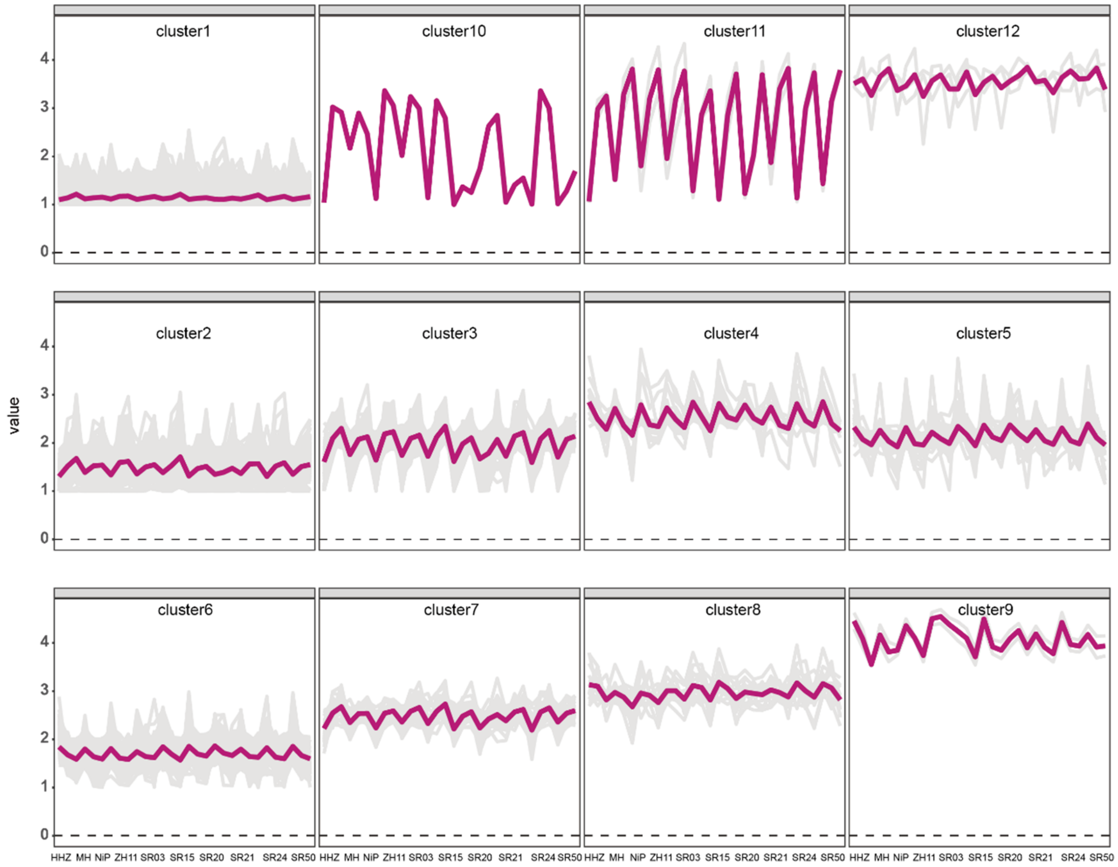

Figure S5. Dynamic analysis of gene expression during seed germination.The gene clusters were represented by red, X-axis indicate different types of rice varieties.

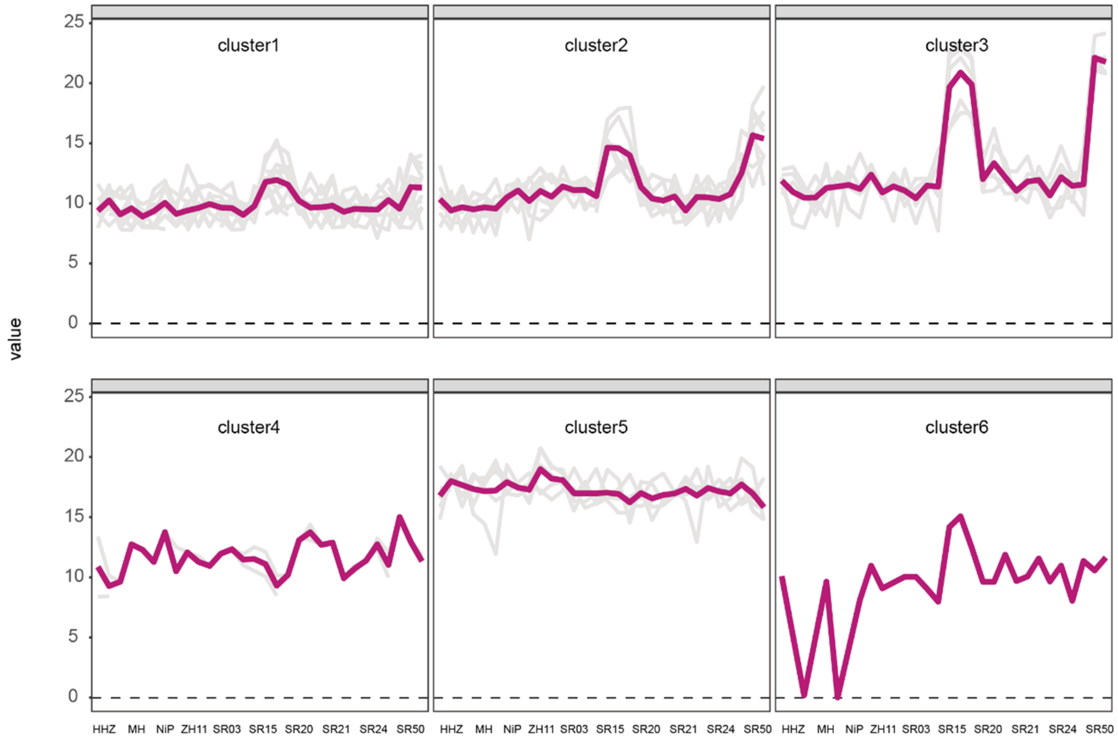

Figure S6. Dynamic analysis of metabolite accumulation during seed germination. The gene clusters were represented by red, X-axis indicate different types of rice varieties.

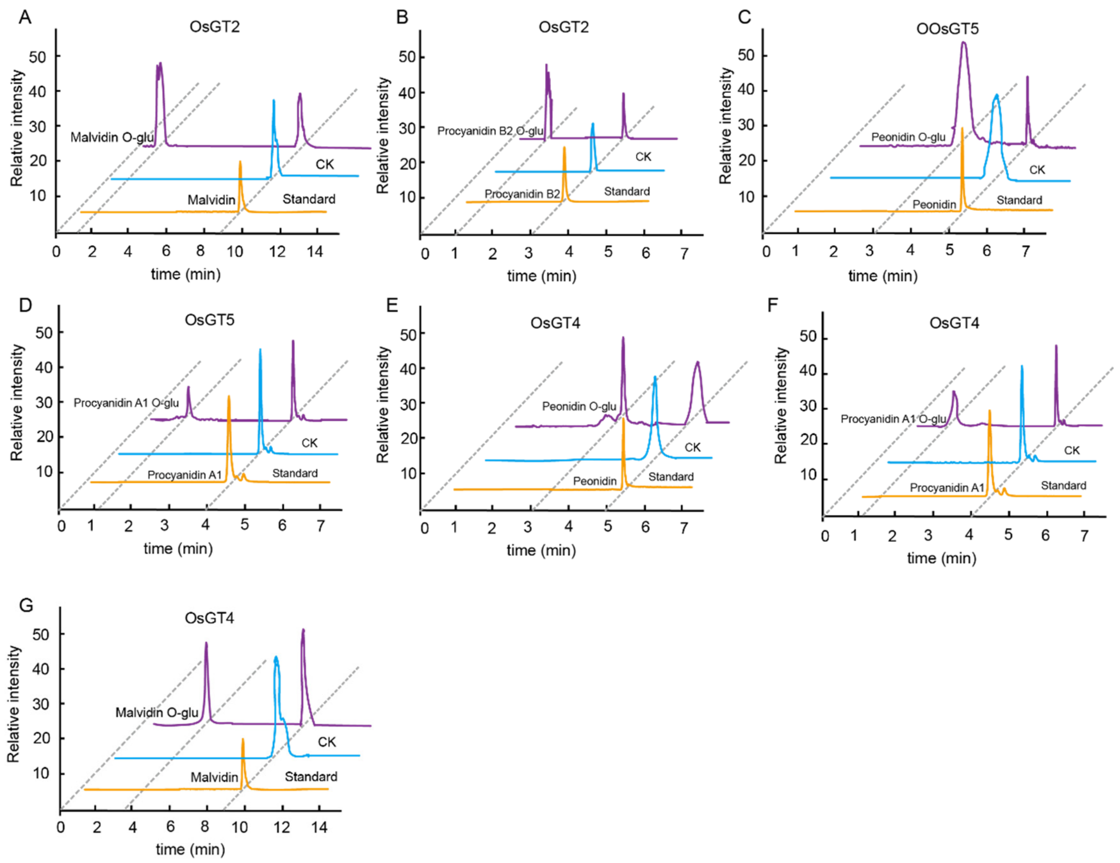

Figure S7. Analysis of *in vitro* enzyme activity of OsGT1 to OsGT5. HPLC chromatograms of the reaction of OsGT1, OsGT2, OsGT3, OsGT4 and OsGT5 with UDP-glucose and different substrates Malvidin (A, G), Procyanidin B2 (B) Peonidin (C, E) and Procyanidin A1 (D, F).

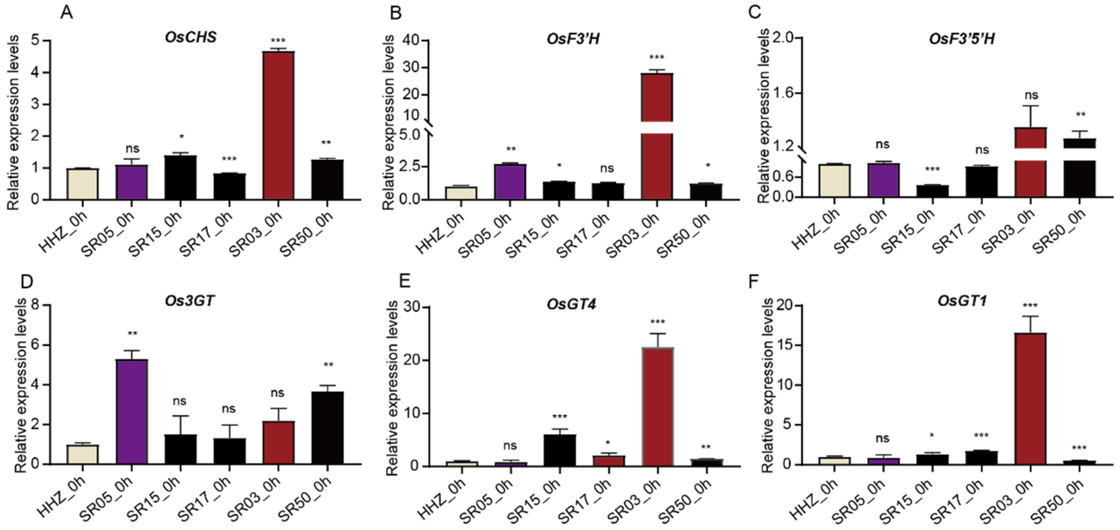

Figure S8. Relative expression levels of structural genes in anthocyanin synthesis in germination seeds of cultivated rice and landrace. (A-F) RT–qPCR results showing the expression levels of *OsCHS, OsF3'H, OsF3'5'H Os3GT, LOC_Os06g18010* (*GT4*) and *LOC_Os07g32630* (GT1) after rice seeds germination 0h. The X-axis refers to different types of rice and the Y-axis is the relative expression. For (A to F), error bars represent the SD of three biological replicates. Asterisks indicate significant differences from the HHZ. according to Student's t-test at *, P<0.05, ***P* < 0.01. ns, not significant.

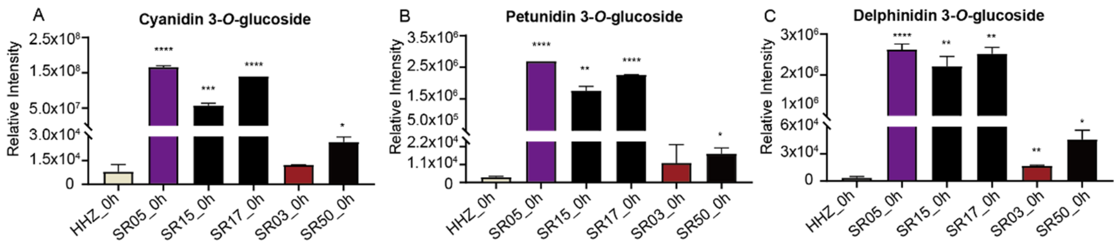

Figure S9. Relative content of anthocyanins in germination seeds of cultivated rice and landrace. The relative content of Cyanidin-3-*O*-glucoside, peonidin *O*-hexoside, Petunidin 3-*O*-glucoside and Delphinidin 3-*O*-glucoside in the cultivar and landrace rice seeds; For (A), error bars represent the SD of two biological replicates. *, $P<0.05$, **, $P<0.01$, ***, $P<0.05$ and ****, $P<0.001$ indicate significant differences.
